# Glomus Tumor in the Left Submandibular Region: A Rare Case Report and Literature Review

**DOI:** 10.1002/cnr2.70113

**Published:** 2025-01-07

**Authors:** Huan Liu, Chengyao Zhu

**Affiliations:** ^1^ Department of General Practice Affiliated Hospital of Hangzhou Normal University Hangzhou China; ^2^ Department of Dermatology the Fourth Affiliated Hospital of School of Medicine, and International School of Medicine, International Institutes of Medicine, Zhejiang University Yiwu China

**Keywords:** glomus tumor, immunohistochemistry, molecular diagnostics techniques, submandibular region, surgical excision

## Abstract

**Background:**

Glomus tumors are rare, benign mesenchymal neoplasms predominantly located in subungual regions of the extremities. Their occurrence in the mandibular region is exceptionally uncommon, presenting unique diagnostic challenges. Only a limited number of submandibular glomus tumors have been documented, leaving their presentation and management largely underexplored.

**Case:**

We presented a case of glomus tumor in the submandibular area of a 60‐year‐old female, which appeared as a purplish‐red lesion. In the absence of characteristic symptoms such as tenderness and cold sensitivity, the lesion was initially misdiagnosed as a pigmented nevus. Histopathological analysis subsequently confirmed the diagnosis as a glomus tumor. Immunohistochemical (IHC) staining further confirmed the tumor's smooth muscle and mesenchymal origins, with positive for Vimentin, SMA, Syn, Actin, Desmin, and CD34. The patient underwent surgical tumor excision with no recurrence after 28 months of follow‐up.

**Conclusion:**

This case underscores the importance of considering glomus tumors in atypical locations and highlights the need for a comprehensive diagnostic approach to prevent misdiagnosis. Surgical excision remains the primary treatment, with extended postoperative surveillance recommended to monitor for recurrence.

## 1 Introduction

Glomus tumors are rare mesenchymal neoplasms that arise from the perivascular modified smooth muscle cells of the glomus body, a specialized structure involved in thermoregulation [1, 2]. These tumors are most commonly found in the subungual regions of the hand, where they present with significant pain and tenderness [3]. However, glomus tumors occurring in the mandibular region are exceptionally rare, with only a limited number of cases documented in the medical literature [4, 5]. This report presents a rare case of a glomus tumor in the left submandibular region, highlighting the diagnostic challenges associated with atypical locations and the importance of comprehensive evaluation. In this case, we provide a detailed discussion of the clinical presentation, diagnostic journey, and challenges in recognizing glomus tumors in unusual locations. We emphasize the need for careful histopathological and immunohistochemical (IHC) analysis to confirm the diagnosis. By presenting this rare case, we aim to raise awareness among clinicians and contribute to a better understanding of glomus tumors in atypical sites.

## 2 Case

A 60‐year‐old female patient presented to the Department of Dermatology at The Fourth Affiliated Hospital of Zhejiang University School of Medicine in April 2022, with a 5‐year history of a purplish‐red nodule in her left submandibular region. The patient reported occasional mild pain but no significant sensitivity to cold or tenderness. The nodule exhibited a very slow growth rate, with no other notable changes over time.

**FIGURE 1:**
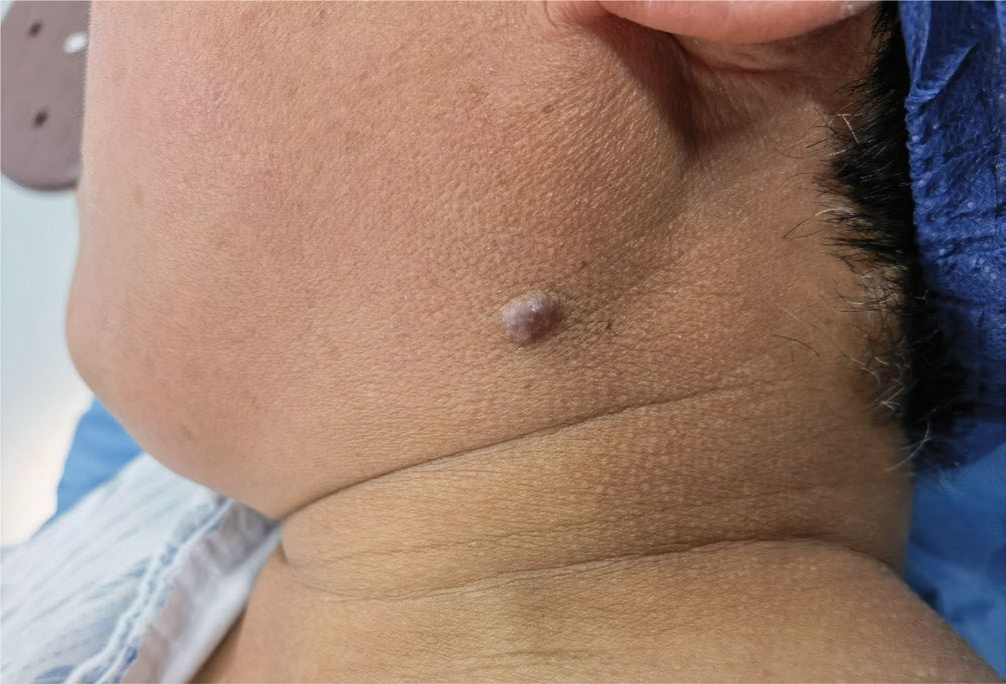
A 0.7 × 0.5 cm^2^ purplish‐red domed nodule located below the left mandibular angle.

Upon examination, the patient had a 0.7 × 0.5 cm^2^, nontender, firm, purplish‐red domed nodule below the left mandibular angle (Figure 1). No pain was induced by tactile stimulation or cold exposure. The patient's medical history was unremarkable. Blood tests, including complete blood count, coagulation profile, and screenings for hepatitis B, syphilis, HIV, and hepatitis C, were all within normal limits.

The lesion was misdiagnosed as a pigmented nevus, a benign skin condition. This misdiagnosis was largely due to its appearance: a nontender, domed, purplish‐red nodule, and the absence of typical glomus tumor symptoms. Consequently, no preoperative imaging, such as B‐mode ultrasound (B‐ultrasound) or magnetic resonance imaging (MRI), was performed. The lesion's location, combined with daily friction while washing the face, raised concerns about potential malignant transformation, prompting excision in the outpatient surgical setting.

**FIGURE 2:**
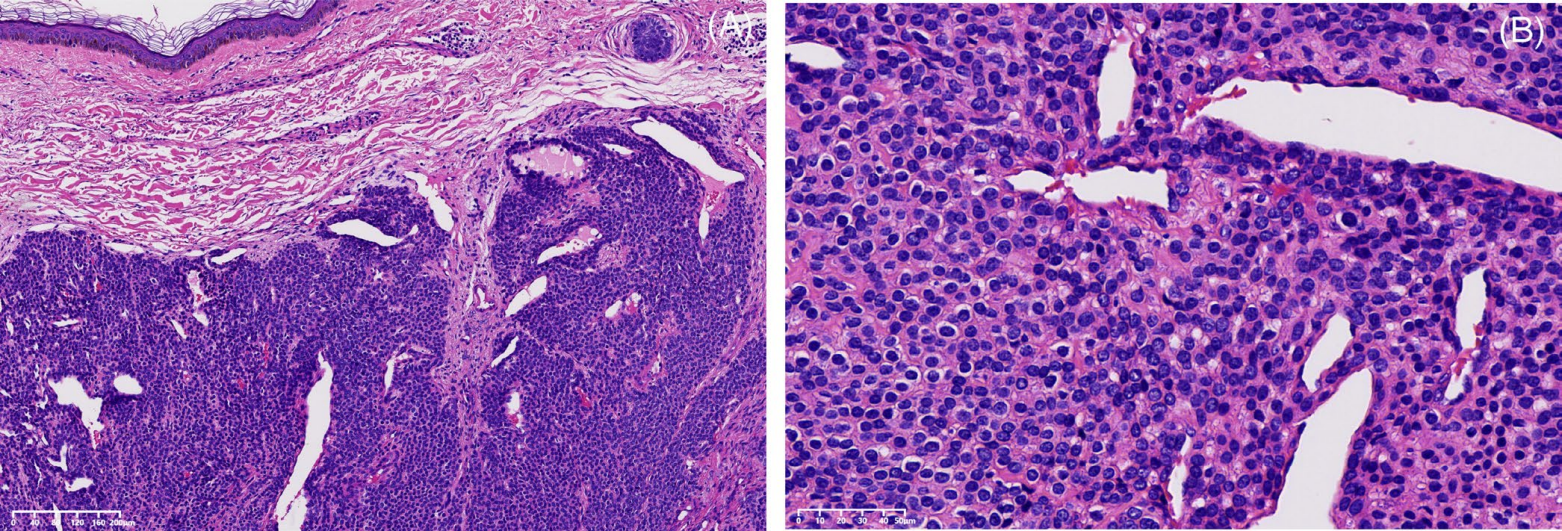
Histopathology demonstrates uniform, small, round cells with centrally located nuclei set within a slightly eosinophilic cytoplasm. Small vessels surrounded by glomus cells, with prominent vascular component, no nuclear atypia or atypical mitotic figure was observed (hematoxylin and eosin) (A; 10×; B; 40×).

**FIGURE 3:**
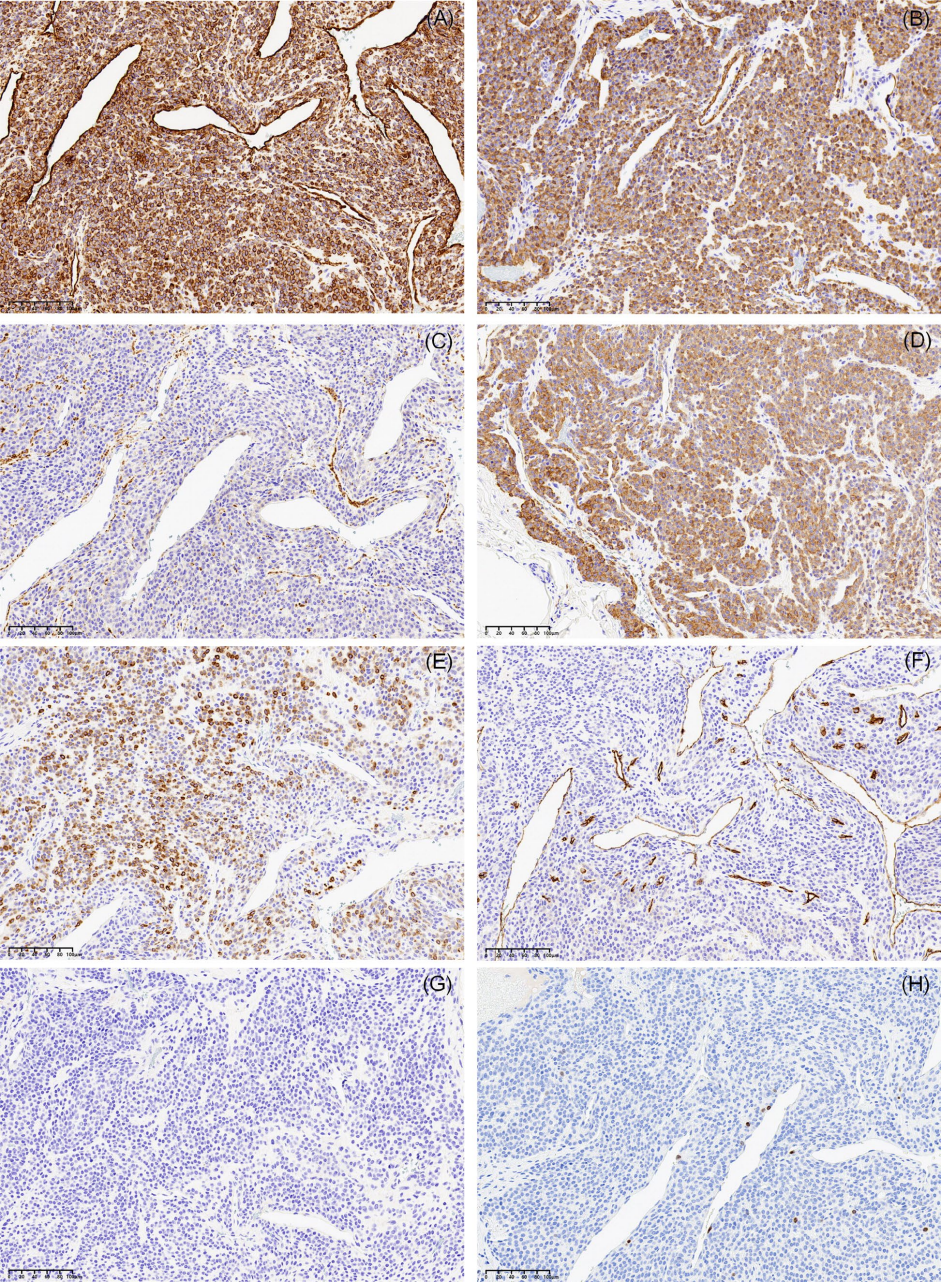
Immunohistochemical staining indicated that the tumor cells were positive for vimentin (A; 20×), SMA (B; 20×), Syn (C; 20×), actin (D; 20×), desmin (E; 20×), CD34 (F; 20×), and negative for CgA (G; 20×). The Ki‐67 (H; 20×) proliferative index is 1% in the glomus tumor.

Histological examination revealed a rich vascular component with round or oval cells arranged in nest‐like or sheet‐like patterns around the blood vessels. Most of their nuclei were small and round within a slightly eosinophilic cytoplasm. Neither nuclear atypia nor atypical mitotic figure was observed. The stromal component exhibited mucoid and hyaline degeneration (Figure 2A,B). The diagnosis was determined as a glomus tumor. IHC staining further confirmed tumor's smooth muscle and mesenchymal origins, with positive for vimentin (Figure 3A), smooth muscle antibody (SMA, Figure 3B), synapsin (Syn, Figure 3C), actin (Figure 3D), desmin (Figure 3E), CD34 (vascular, Figure 3F), while negative for and chromogranin A (CgA, Figure 3G). The Ki‐67 mitotic index labeled 1% of the tumor cells (Figure 3H). These markers support the diagnosis by differentiating glomus tumors from other neoplasms with similar features.

The patient has been regularly followed up for 28 months, and there have been no indications of recurrence. She remains asymptomatic, and no complications have been reported.

## 3 Discussion

Glomus tumors are relatively rare in the clinic. They are vascular neoplasms consisting of benign glomus cells originating from the glomus body [6]. The glomus body is a specialized arteriovenous anastomosis that plays a crucial role in thermoregulation. These structures are particularly concentrated in areas like the fingers and toes, which explains the common occurrence of glomus tumors in these regions [7]. These tumors can present as solitary or multiple lesions. Solitary tumors are majorly located in the digits, especially in the subungual region, and show a strong female predominance [7]. Although glomus tumors are most commonly found in fingers and toes, they can also occur in extradigital sites, such as arm [6], knee [8], stomach [9], duodenum [10], pulmonary [11], and other areas. However, their occurrence in the mandibular region is exceptionally rare, introducing unique diagnostic challenges.

A comprehensive review of the literature reveals only a limited number of documented cases, each presenting its own set of diagnostic challenges and clinical intricacies. Recently published case reports regarding glomus tumors of the face and submandibular area were reviewed [4, 5, 12, 13, 14, 15, 16, 17, 18, 19, 20, 21, 22, 23, 24, 25]. The patient characteristics of all 18 cases (including our case) are shown in Table 1. Only two cases of glomus tumors of the submandibular area (both with multiple lesions, not limited to the submandibular region) have been reported, emphasizing the rarity of solitary glomus tumors in the submandibular region. A predominant number of cases (*n* = 7) reported tumors occur in the eyelid region. The neoplasms developed in nine males and nine females (male‐to‐female ratio, 1:1), and patient ages spanned a broad spectrum, ranging from 8 to 74 years. Notably, the predominant clinical manifestation was a painful mass or nodule, yet some tumors remained asymptomatic. The interval between the tumor's onset and its subsequent treatment demonstrated considerable variation, with certain patients seeking medical attention only after several years had passed. Surgical excision is the most common treatment, and follow‐up data for seven of the documented cases show no recurrence within 6 months to 3 years. The intravascular lesions without overlying skin abnormality have not been included.

**TABLE 1:** Summary of clinical and outcome features of glomus tumors in the facial and submandibular regions.

Year	Authors	Age (yr)	Sex	Site	Symptom	Duration of the glomus tumor (yr/m)	Treatment	Follow‐up time	Outcomes
1940	Kirby [12]	30	M	Left lower eyelid	Painful mass	8 yr	Surgery	1 yr	No recurrence
1963	Mortada [13]	18	F	Left upper eyelid	Tender swelling	5 yr	Surgery and radiotherapy	2 yr	No recurrence
1965	Jensen [14]	47	M	Left upper eyelid	Slightly tender nodule	7 yr	Surgery	6 m	No recurrence
1976	Charles et al. [15]	17	F	Right side of face, eyelid, anterior orbit, and palate	Painless patches	NA	Surgery	NA	NA
1985	Saku et al. [16]	45	M	Left cheek	Painless nodular	6 m	Surgery	NA	NA
1993	Saxe et al. [17]	39	M	Left upper eyelid	Painless mass	28 yr	Surgery	NA	NA
1993	Saxe et al. [17]	18	F	Left lower eyelid	Painless swelling	1 yr	Surgery	NA	NA
1996	Savaci et al. [18]	55	F	Left buccal mucosa	Painful mass	6 m	Surgery	NA	NA
2000	Yu et al. [4]	54	F	Left mandibular area, lip, anterior buccal mucosa	Painless neoplasm	30 yr	Surgery	NA	NA
2004	Lai et al. [19]	45	F	Right upper eyelid	Painless nodule	12 m	Surgery	18 m	NA
2008	Ma et al. [20]	23	F	Face, right auricle, right arm, and right leg	Painless nodule	2 yr	Biopsy	NA	NA
2011	Yoo et al. [5]	55	M	Right parotid region and submandibular	Painful masses	NA	Surgery	NA	NA
2012	Veros et al. [21]	24	M	Left cheek	Painful nodule	4 yr	Surgery	9 m	No recurrence
2014	D'Antonio et al. [22]	74	M	Forehead	Painful nodule	NA	Wide surgical excision	3 yr	No recurrence
2015	Yazdani et al. [23]	8	M	Right malar, buccal and infraorbital spaces	Painful, severe swelling	1 yr	Surgery	NA	NA
2019	Ning et al. [24]	49	F	Left zygomatic region	Painful nodule	10 yr	Surgery	2 yr	No recurrence
2021	Panickar et al. [25]	30	M	Right lower lid	Painless swelling	6 m	Surgery	2 yr	No recurrence
	Our case	60	F	Left submandibular area	Painful papule	5 yr	Surgery	28 m	No recurrence

Abbreviations: F, female; M, male; m, months; NA, not available; yr, years.

Clinically, glomus tumors can be mistaken for other benign lesions, such as pigmented nevi, due to their similar appearance. Typically, glomus tumors present as blue to purple vascular nodules [26], and the size of these benign tumors is usually less than 2 cm in diameter [27]. Pigmented nevi, commonly referred to as moles, are benign skin lesions resulting from a local proliferation of pigment cells (melanocytes). They typically manifest as round or oval‐shaped, single‐colored lesions ranging from skin‐colored to dark brown, and they are usually of uniform size, with some present since birth known as congenital nevi [28]. However, their origins, histopathological features, and clinical presentations are distinct.

In this study, the misdiagnosis was further compounded by the lesion's deviation from the typical clinical manifestations of extradigital glomus tumors, notably its lack of tenderness and unresponsiveness to cold stimuli. Typically, glomus tumors exhibit a classic triad of spontaneous pain, hyperalgesia to digital pressure, and sensitivity to cold [26]. Furthermore, physicians' lack of clinical experience on glomus tumors contributes to the potential for misdiagnosis.

To improve diagnostic accuracy, it is crucial to employ multiple imaging modalities to confirm atypical glomus tumors and prevent misdiagnosis. B‐ultrasound is particularly valuable in providing real‐time imaging, which can be crucial in differentiating glomus tumors from other superficial lesions. Given its accessibility and non‐invasive, B‐ultrasound is often recommended as the first‐line imaging modality for suspicious cases [29]. In addition to B‐ultrasound, auxiliary imaging for glomus tumors includes x‐ray and MRI. Despite these available tools, misdiagnosis remains common, primarily due to limited familiarity among primary physicians with the typical symptoms and tests for glomus tumors [7]. In our study, the initial diagnosis identified the lesion was a pigmented nevus, leading us to forego B‐ultrasound and MRI evaluations. This oversight highlights a limitation in our diagnostic approach, which we endeavor to rectify in subsequent clinical practices.

Even though glomus tumors are typically benign, those that are deep‐seated have demonstrated a heightened malignancy risk [22]. Misdiagnosis might result in unsuitable treatments, potentially worsening the condition or causing unneeded complications. This emphasizes the importance of thorough clinical and histopathological evaluations for the purpose of accurate diagnosis and treatment, in cases involving atypical lesions [30].

The histopathological findings in our case revealed typical features of a glomus tumor, as illustrated in Figure 2. Our findings reinforce the characteristic IHC profile observed in glomus tumors, marked by positive staining for vimentin, SMA, actin, and desmin, which underscore the tumor's smooth muscle and mesenchymal origins [31, 32, 33]. The presence of CD34 further corroborates the vascular nature of these tumors [32]. Additionally, the low Ki‐67 index supports the benign nature of the tumor, aligning with the typical profile of most glomus tumors [33].

In our study, we observed a unique differentiation pattern within the glomus tumor, characterized by the absence of CgA staining and positive expression of Syn. This finding contrasts with the majority of existing literature, where Syn expression is typically not reported (or negative) in glomus tumors. Syn is generally recognized as a marker associated with neural and neuroendocrine differentiation, suggesting that our case may represent a variant or atypical differentiation pathway. Interestingly, Syn positivity has only been documented in hepatic and gastric glomus tumors [34, 35].

Furthermore, the absence of CgA staining is consistent with existing literature, where CgA is typically absent in glomus tumors, aiding in their distinction from neuroendocrine tumors [36]. These findings highlight the critical importance of employing a comprehensive IHC panel to ensure accurate characterization of glomus tumors, particularly in atypical locations. While our findings are largely consistent with the existing literature on glomus tumors, they also introduce a novel aspect concerning neuroendocrine marker (Syn). These observations emphasize the need to consider potential variants of glomus tumors that may display mixed differentiation patterns, warranting further investigation into the existence and characteristics of such variants.

The differential diagnosis of glomus tumors is pivotal for guiding therapeutic strategies and determining prognosis. Originating from the glomus body—which comprises glomus cells, vascular cells, and smooth muscle cells—these tumors are classified into three subtypes: glomus tumors, glomangiomas (or glomuvenous malformations), and glomangiomyomas, each reflecting the involvement of the different cellular components of the glomus body [37]. ① Glomangiomas, akin to glomus tumors, are marked by a pronounced vascular component and typically present as multifocal, painful blue‐purple lesions. Histologically, they are characterized by dilated vascular spaces, in contrast to the solid appearance of glomus tumors. Immunohistochemically, they are positive for vimentin and α‐SMA but negative for desmin, von Willebrand factor, and S‐100 [38]. ② Glomangiomyomas, predominantly composed of smooth muscle cells, are histologically distinct and are further categorized into regional, disseminated, and congenital plaque‐like subtypes. ③ Differentiation from GLI1‐altered mesenchymal tumors presents a diagnostic challenge due to their histopathological similarity to glomus tumors. However, GLI1‐altered neoplasms can be distinguished by the absence of the classic triad. GLI1‐altered mesenchymal tumors often manifest in the head and neck, particularly within the tongue [39]. IHC profiling is essential, as glomus tumors typically express markers such as vimentin, SMA, calponin, actin, desmin, and CD34, while GLI1‐altered mesenchymal tumors may exhibit positivity for vimentin, S100, CD56, cyclinD1, and negativity for SOX10, SMA, melan‐A, HMB‐45, synaptophysin, and a variety of other markers, indicating significant immunospectrum shifts [40]. ④ Malignant glomus tumors are exceedingly rare vascular malignancies of unclear etiology, which are believed to arise de novo or from the malignant transformation of a benign glomus tumor [41]. They exhibit nuclear pleomorphism and increased mitotic activity, and their identification is enhanced by immunohistochemistry and molecular diagnostics. ⑤ Vascular neoplasms like hemangiomas and angiosarcomas are distinguishable by their unique growth patterns and IHC markers.

Recent RNA sequencing has identified a novel NOTCH‐MIR143 fusion gene in over half of glomus tumors, irrespective of the tumors' malignancy or anatomical location. Detection of NOTCH‐MIR 143 fusion gene is instrumental for correct diagnosis and differential diagnosis [42]. Familial occurrences of glomus tumors suggest a genetic predisposition, with mutations in genes such as NOTCH3 and PDGFRB implicated in some cases, though the complete genetic landscape remains incompletely understood [42]. Approximately 6% of glomus tumors with uncertain malignant potential exhibit the BRAF V600E mutation [43], and the majority are positive for PDGFRB staining [42]. EWSR1 (22Q12) rearrangement may be present in isolated cases [44]. The elucidation of molecular mechanisms in glomus tumors paved the way for targeted therapies. For instance, tumors with aberrant NOTCH3 signaling may respond to NOTCH pathway inhibitors [42].

While surgical excision remains the gold standard for treating glomus tumors, GLI1‐altered mesenchymal tumors could respond to targeted therapies, particularly those inhibiting the Hedgehog signaling pathway [45]. Therefore, incorporating molecular diagnostics into the diagnostic workflow is crucial for informed clinical decision‐making and the development of personalized treatment plans.

Managing glomus tumors in the mandibular region comes with unique challenges. Previous series have reported a 10%–30% recurrence rate following the resection of glomus tumors [46]. The golden standard treatment for glomus tumors is complete surgical excision [47, 48]. Depending on the anatomical location of the tumor, we performed excision under local anesthesia. Although glomus tumors, as mesenchymal vascular neoplasms, are typically prone to bleeding during surgery, this patient did not experience significant intraoperative bleeding. The tumor was successfully and entirely removed. During follow‐up, the patient experienced complete recovery with significant relief of symptoms. At the 28‐month follow‐up, there was no evidence of recurrence. Therefore, precise diagnosis and diligent follow‐up are essential for effectively managing glomus tumors, particularly in atypical locations.

## 4 Conclusion

This research presents a rare case of a solitary glomus tumor in the left submandibular region, adding to the limited documentation of glomus tumors in the facial and submandibular areas, bringing the total number of cases to 18. It emphasizes the need for clinicians to remain vigilant for extradigital glomus tumors, especially when evaluating nontender, temperature‐insensitive skin lesions. Surgical excision remains the primary treatment, with a strong emphasis on extended postoperative surveillance to monitor for recurrence. Further research is essential to enhance diagnostic and treatment approaches, particularly for glomus tumors in atypical locations.

## Author Contributions

**Huan Liu:** writing – original draft, conceptualization, investigation, project administration. **Chengyao Zhu:** writing – review and editing, data curation, conceptualization.

## Ethics Statement

The authors have nothing to report.

## Conflicts of Interest

The authors declare no conflicts of interest.

## Data Availability

The data are available from the author upon reasonable request.
